# PHENOTYPES OF COGNITIVE FUNCTION IN THE CONTEXT OF COMORBIDITIES

**DOI:** 10.1093/geroni/igad104.0655

**Published:** 2023-12-21

**Authors:** Yaguang Zheng, Weiyu Mao, Jing Wang

## Abstract

While a growing body of evidence has shown that the prevalence of cognitive impairment varies depending on the population, limited research has explored the complex relationships among different cognitive function domains or changes in cognitive functions in the context of comorbidities (e.g., diabetes and stroke). The objective of this symposium is to address knowledge gaps by demonstrating these complex relationships in the context of comorbidities. Zheng and colleagues discuss the prevalence of mild cognitive impairment and dementia in adults with diabetes using nationally representative data from a large-scale dataset. Zhu et al. explore the inference of changes in cognitive function and relationships among cognitive function domains using a novel phenotypic network approach. Further, Li et al. use a similar phenotypic network approach to identify the dynamic associations across cognitive, physical, psychological function, and quality of life among Chinese patients with acute ischemic stroke. Nan Wang & colleagues discuss factors associated with the perceived risk of Alzheimer’s disease and related dementias (ADRD) and how the perceived risk of ADRD is related to cognitive function. Following the paper presentations, Dr. Jing Wang will lead a discussion. The novel finding from this symposium will provide a more comprehensive understanding of how cognition changes over time and how interventions could target multiple domains or be tailored to the varied medical comorbid conditions to improve overall cognitive functions.

